# The Design and Development of an Omni-Directional Mobile Robot Oriented to an Intelligent Manufacturing System

**DOI:** 10.3390/s17092073

**Published:** 2017-09-10

**Authors:** Jun Qian, Bin Zi, Daoming Wang, Yangang Ma, Dan Zhang

**Affiliations:** 1 School of Mechanical Engineering, Hefei University of Technology, 193 Tunxi Road, Hefei 230009, China; qianjun@hfut.edu.cn (J.Q.); denniswang@hfut.edu.cn (D.W.); myg_ballack@mail.hfut.edu.cn (Y.M.); 2 Lassonde School of Engineering, York University, 4700 Keele Street, Toronto, ON M3J 1P3, Canada; dzhang99@yorku.ca

**Keywords:** omni-directional mobile robot, localization, extended Kalman filter (EKF), intelligent manufacturing system, design

## Abstract

In order to transport materials flexibly and smoothly in a tight plant environment, an omni-directional mobile robot based on four Mecanum wheels was designed. The mechanical system of the mobile robot is made up of three separable layers so as to simplify its combination and reorganization. Each modularized wheel was installed on a vertical suspension mechanism, which ensures the moving stability and keeps the distances of four wheels invariable. The control system consists of two-level controllers that implement motion control and multi-sensor data processing, respectively. In order to make the mobile robot navigate in an unknown semi-structured indoor environment, the data from a Kinect visual sensor and four wheel encoders were fused to localize the mobile robot using an extended Kalman filter with specific processing. Finally, the mobile robot was integrated in an intelligent manufacturing system for material conveying. Experimental results show that the omni-directional mobile robot can move stably and autonomously in an indoor environment and in industrial fields.

## 1. Introduction

Intelligent manufacturing and logistics have motivated the development of mobile robot technology in recent years [1]. Intelligent factories have been increasingly demanding autonomous mobile robots to fulfill various movement tasks [2,3]. In consideration of a complicate working environment, mobile robots need to be flexible, adaptive, and safe [4,5]. Consequently, traditional mobile robots based on differential drive with large turning radii [6] are not satisfactory.

There are many kinds of omni-directional mobile robots that have been developed and produced [7]. Two typical types of wheels can realize the omni-directional movement, i.e., a steerable wheel and an omni-directional wheel [8]. Both of them have been used for mature products, including Adept Seekur and KUKA omniRob robots. The steerable wheel has its own rotational mechanism, which can change its steering angle actively [9], while the omni-directional wheel uses a special wheel structure for flexible movement, examples of which include the Swedish wheel [10] and the Mecanum wheel. Swedish wheels have been widely used on small moving platforms, especially on robot soccers. Owing to its ideal pushing force, simple mechanism, and easy control performances, Mecanum wheels are more appropriate for carrying heavy goods in the industrial environment [11]. Unfortunately, Mecanum wheels also have several disadvantages. The special wheel structure allows for the lateral movement degree-of-freedom (DOF), but the rotation of the rollers around the Mecanum wheel will result in the slippage of the mobile robot [12]. This slippage is difficult to be modeled and calculated. Meanwhile, the gap between the two adjacent rollers causes a periodical vibration in the moving platform, which obviously affects movement accuracy and stability.

To deal with the accumulate movement errors and estimate the positions of mobile robots, many efforts have been implemented by integrating exteroceptive sensors and using data fusion algorithms to acquire accurate localization results. The natural environment is usually changed by mounting featured structural objects for exact measurements, for example, passive reflectors [13], artificial landmarks [14], and the Radio Frequency Identification (RFID) technique [15]. However, infrastructure reconstructions are not universal because they affect flexibility, especially for intelligent factories. Therefore, it is more important to use on-board sensors to measure the natural environment. The inertial navigation system is appropriate for most mobile platforms, while the high performance restricts its hardware cost [16]. With the development of the Microsoft Kinect sensor and its successful application in the field of entertainment, the integration of the Kinect sensor and other on-board sensors can also be used on mobile robots [17]. The methods and experiments of data fusion on omni-directional mobile robots have been widely researched [18,19]. However, further comprehensive works should be completed since the industrial fields are more complicated and diversified.

The objective of intelligent manufacturing is a cyber-physical system (CPS), which is made up of physical entities equipped with perception, processing, and decision-making abilities [20]. There are two flows in the intelligent manufacturing system (IMS), i.e., material flow and information flow. In order to convey materials flexibly and efficiently when facing multi-station and multi-task applications, intelligent mobile robots need to be specially developed so as to satisfy the whole system and the industrial environment. Meanwhile, the data exchange between physical entities relies on unified interfaces and communication standards [21], which are also difficult since many traditional physical entities need upgrades. From this point of view, intelligent mobile robots oriented to IMS are very important components of smart factories and smart logistics.

In this paper, we focus on designing and developing an omni-directional mobile robot with four Mecanum wheels from the systematic point of view. The whole process resembles the design of other intelligent systems or microsystems [22]. Firstly, modularized wheels with a vertical suspension mechanism are designed to realize the flexible and steady movement of the mobile robot. The multi-layer mechanical structure of the body is utilized to be convenient for easy upgrades and different applications. Secondly, the control system needs to give consideration to the reliability, safety, and intelligence of the mobile robot. Therefore, a lower-level controller uses an industrial grade controller for motion control, and a higher-level controller uses an embedded industrial PC for multi-sensor data processing. In order to navigate autonomously in unknown semi-structured indoor environment, data from wheel encoders and a Kinect visual sensor are fused to localize the mobile robot. Finally, the mobile robot is applied in an IMS for conveying materials by experimental tests. It is expected that the mobile robot provide a solution of intelligent mobility in smart factories with a low cost and relative high accuracy.

The rest of this paper is organized as follows: Section 2 and Section 3 describe the mechanical and control systems of the mobile robot in detail, respectively. Section 4 presents the positioning system of the mobile robot by using a simplified extended Kalman filter. Section 5 shows the experimental results of localization and mapping. Finally, conclusions are presented in Section 6.

## 2. Mechanical System of the Mobile Robot

**Figure 1:**
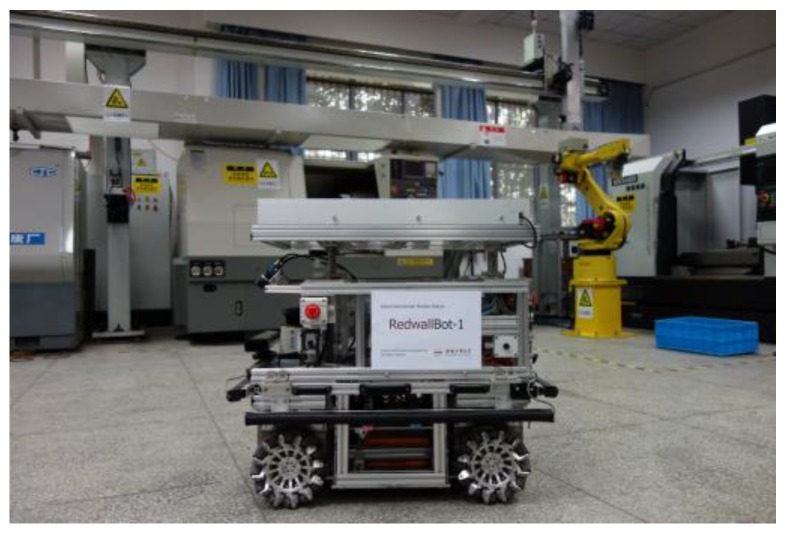
Omni-directional mobile robot RedwallBot-1.

Since the environment of intelligent manufacturing systems are commonly favorable indoor sites crowded with equipment and materials, an omni-directional mobile robot is required to move flexibly in narrow spaces. In this paper, a four-wheel independent driven structure by using the advantages of Mecanum wheels is designed [23]. The mobile robot is named RedwallBot-1 and shown in Figure 1. In order to ensure expansibility and versatility, multi-layer mechanical modules and a modular wheel structure are used for its body.

### 2.1. Modular Wheel Structure

The small solid rollers on the circumference of each Mecanum wheel are arranged with gaps, which may bring periodical vibrations in the mobile platform. One solution is to use elastic wheel hubs to absorb the vibration [24]. Another solution is to add suspension mechanisms on the whole Mecanum wheel [25]. Different suspension mechanisms have been designed to keep four Mecanum wheels in contact with the ground. Traditional suspension mechanisms use a link with dampers for shock absorption, which is similar to the damping of automobiles. However, a mobile robot using this suspension has variable distances between longitudinal or lateral wheels.

**Figure 2:**
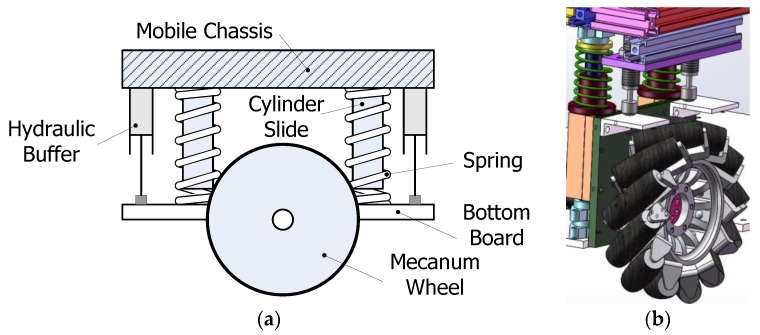
Modular wheel structure with suspension. (**a**) The components of the modular wheel; (**b**) 3D model of the modular wheel.

A modular wheel structure with vertical suspension mechanisms is designed as shown in Figure 2. The Mecanum wheel on the bottom board is connected with the mobile chassis compliantly. The connectors include two cylinder slides and linear bearings, while the shock absorbers include two springs and buffers, respectively. Therefore, the Mecanum wheel can regulate its position vertically and passively with springs according to the loads and the ground topography. In the design, two hydraulic buffers are used to absorb the damping energy quickly. Both the springs and the buffers can be replaced easily according to different loads on the mobile platforms.

### 2.2. Multi-Layer Mechanical Modules

**Figure 3:**
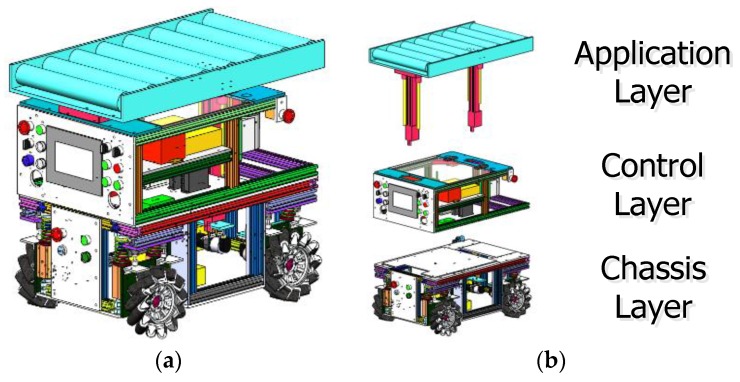
The mechanical components of the robot body. (**a**) 3D model of the mobile body; (**b**) three independent layers.

The body of RedwallBot-1 possesses a three-layered mechanical structure from bottom to top, i.e., the chassis layer, the control layer, and the application layer. Two adjacent layers are connected with screw bolts, which can be easily assembled and disassembled. Figure 3 shows the mechanical components of the robot body. Both the chassis layer and the control layer constitute the main body of the mobile platform. General specifications of RedwallBot-1 are shown in Table 1.

(1). The Chassis Layer

This layer contains the movement mechanism and the power source. Each modular wheel is connected with a DC servomotor and a harmonic reducer along the rotational axis of the Mecanum wheel. Each DC servomotor is a Maxon RE40 with an output power of 150 W. The ratio of the harmonic reducer is 50:1. Four modular wheels are arranged symmetrically along the longitudinal center axis of the chassis. Meanwhile, a 48 V 40 Ah lithium battery is used to supply power for the mobile platform.

(2). The Control Layer

This layer consists of the control system, the on-board sensory suite, and the power converters. The control system includes the controller, motor drives, an internet router, and a touch screen. An Elmo CEL-10/100 DC drive is used for each Maxon servomotor, which has a built-in PID control function [26,27,28]. According to the expected input speed of the drive and the feedback from the encoder at the end of the servomotor, the PID parameters can be regulated manually to obtain ideal dynamic responses. A touch screen is used to show the parameters and status graphically. Moreover, a handheld control box is made to manually operate the mobile platform by using a 3-axis joystick based on three potentiometers.

(3). The Application Layer

This layer has many selections to meet different needs, e.g., a light-weight robot manipulator and a fork arm. RedwallBot-1 is oriented to convey materials for an intelligent manufacturing system, so a linear transfer line is designed to carry a rectangular container. Several transfer rollers are placed horizontally on the top of the mobile chassis. Among those rollers, one electric transfer roller rotates with other passive rollers together using chain transmission. In order to adapt different heights at several work stations, two electric cylinders are placed under the transfer line to offer vertical propulsion.

## 3. The Control System of the Mobile Robot

### 3.1. Control Hardware

**Figure 4:**
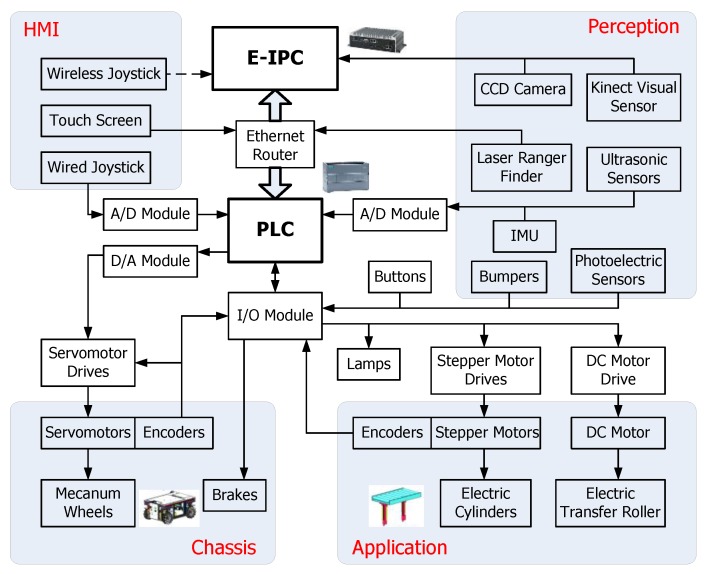
Block diagram of RedwallBot-1’s control system.

In order to process considerable sensory data and manipulate the mobile robot autonomously, the control system of RedwallBot-1 is made up of two layers. The lower-level and higher-level controllers use a programmable logic controller (PLC) and an embedded industrial PC (E-IPC), respectively. The two-level controllers communicate with each other with Ethernet. Figure 4 shows the whole control system of the mobile robot. The peripheral devices are classified into four modules, i.e., mobile chassis, special application, perception, and human machine interface (HMI).

The lower-level control system is used to provide the function of mobility. In order to control many devices reliably and flexibly, a PLC with a Siemens S7-1217C CPU was selected as the lower-level controller. Several extended modules for the PLC were also used, which include an I/O module, an A/D module, and a D/A module.

The Mecanum wheel-based omni-directional mobile robot uses the motion model of differential drive. Therefore, each driving wheel is rotated under the velocity control. The voltage signal is sent to each servomotor drive to control the wheel. By changing the ratios of 4-channel voltage signals, the mobile robot can move in different motion modes including longitudinal, lateral, diagonal, and rotational movements. The signal of each encoder installed at the rear of the servomotor is sent to both the related drive and the PLC simultaneously. Thus, the PLC can calculate the movement of the mobile chassis based on odometry. Meanwhile, two electronic cylinders are controlled synchronously to lift the transfer rollers up and down.

The higher-level controller is an embedded industrial PC with the type of Advantech ARK-2150F. It acquires multi-sensor data and processes data in real time. Therefore, localization, map building, and path planning of the mobile robot can be implemented.

### 3.2. Perception

**Figure 5:**
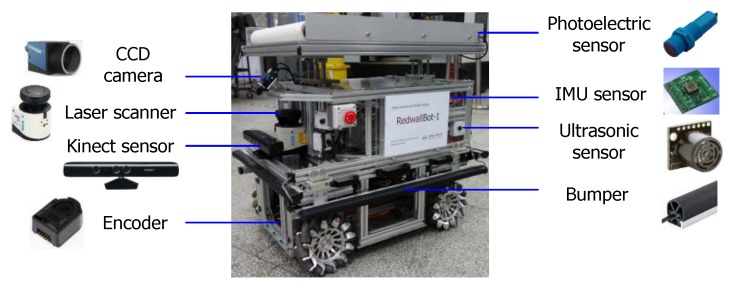
Assignment of the on-board sensors.

The on-board sensory suite mainly includes a vision sensor, a laser scanner, ultrasonic sensors, and inertial measurement sensors (IMUs). In front of the mobile robot, a CCD camera is installed obliquely so as to follow the lane line on the ground. Meanwhile, a Kinect visual sensor is utilized to perceive the nearby environment by integrating the color image and depth image. A laser scanner (SICK LMS111 laser ranger finder) is used to measure the obstacles of the 2D environment accurately. Eight Maxbotix ultrasonic sensors are installed on four faces of the robot averagely. Several low cost sensors are used to provide basic perceptions, such as bumpers and photoelectric sensors. The laser scanner, ultrasonic sensors, and bumps together make up the safety system for obstacle avoidance. Figure 5 shows the on-board sensors and their assignment on the mobile robot.

### 3.3. Software Architecture

**Figure 6:**
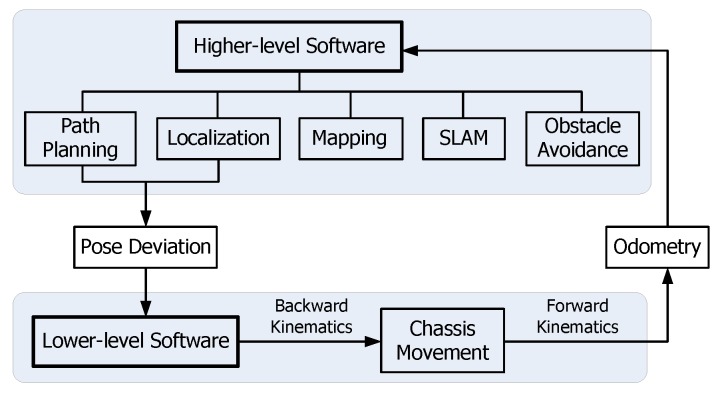
Schematic diagram of the control software.

The two-level control software is designed according to the hardware structure. Figure 6 shows the whole control diagram and their data exchange.

#### 3.3.1. Lower-Level Software

**Figure 7:**
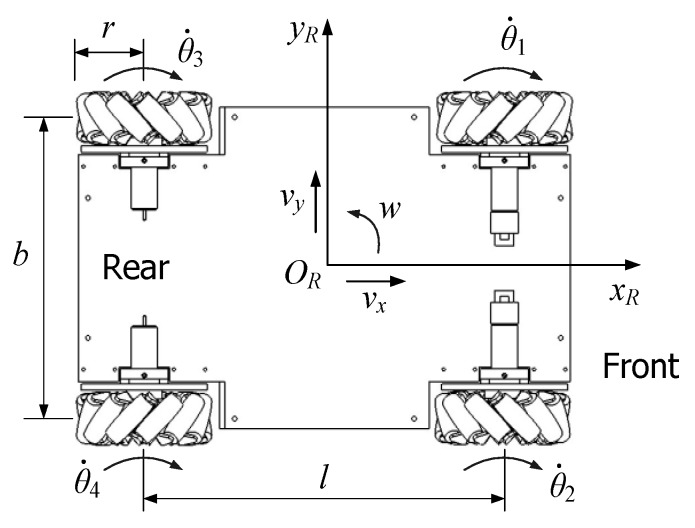
The distribution of the four wheels and their movements.

The lower-level software focuses on motion control and the movement safety of the mobile robot. Figure 7 shows the distribution of four Mecanum wheels on the mobile chassis from the vertical view. The forward and backward kinematics calculations are given in Equations (1) and (2), respectively.

(1) [vxvyw]=πr720[11111−11−1−1d1d−1d1d][θ˙1θ˙2θ˙3θ˙4]

(2) $$\left[\begin{array}{c} {\dot{\theta }}_{1}\\ {\dot{\theta }}_{2}\\ {\dot{\theta }}_{3}\\ {\dot{\theta }}_{4} \end{array}\right]=\frac{180}{\pi r}\left[\begin{array}{ccc} 1 & 1 & -d\\ 1 & -1 & d\\ 1 & -1 & -d\\ 1 & 1 & d \end{array}\right]\left[\begin{array}{c} v_{x}\\ v_{y}\\ w \end{array}\right]$$

where *v_x_* and *v_y_* are the longitudinal and the lateral velocities of the mobile robot, and *w* is the angular velocity. They constitute the velocity vector ***V*** = (*v_x_*, *v_y_*, *w*)^T^ of the mobile robot. The dot above *θ_i_* indicates the rotating speed of the *i*-th wheel (*i* = 1, 2, 3, 4) in deg/s. In addition, *r* is the radius of the Mecanum wheel. In Figure 7, *l* and *b* are the distances between the longitudinal and the lateral wheels, respectively. *d* is one 720th of the sum of *l* and *b*, so *d* = (*l* + *b*)/720.

In order to control the movement of the mobile chassis, the velocity or the pose deviation is firstly converted into the rotating speed or angle of each wheel based on the backward kinematics. Meanwhile, according to data from the four wheel encoders, the robot’s velocity is estimated based on the forward kinematics.

#### 3.3.2. Higher-Level Software

The higher-level software processes the sensory data and sends the control orders to the mobile robot for navigation. Two exteroceptive sensors are mainly used, i.e., a CCD camera and a Kinect sensor. By using OpenCV (Open Source Computer Vision Library), the color image from each visual sensor can be conveniently processed. When tracking a fixed path on the ground, the lane line is extracted firstly and fitted with a straight line or circular curve. Then, the deviations of the position and the heading angle are calculated and the results are sent to the lower-level software to control the mobile robot by using a PID control algorithm. By using a preview control, the mobile robot finds one destination position on the lane line. According to the movement characteristic of the mobile robot shown in Equation (1), an arc trajectory is dynamically planned and traced. Therefore, the geometric center of the mobile robot can keep moving along the lane line. If the mobile robot navigates based on a free path planned by the higher-level controller, multi-sensor data fusion algorithm will be used for localization. Since the mobile robot has the omni-directional mobility, the obstacle avoidance is flexible and diverse. In order to shorten the development cycle, the higher-level control system uses Linux and open-source Robot Operating System (ROS) [29,30]. OPC Unified Architecture (UA) specification [31] is a platform-independent communication standard for industrial applications. Therefore, ROS-based higher-level control software could access data with Siemens PLC and other field devices using OPC UA [32]. The lower-level controller, as an OPC UA server, communicates with E-IPC and other clients through wired or wireless Ethernet. Therefore, the mobile robot could be competent for complicated production systems.

### 3.4. Integration with an Intelligent Manufacturing System

**Figure 8:**
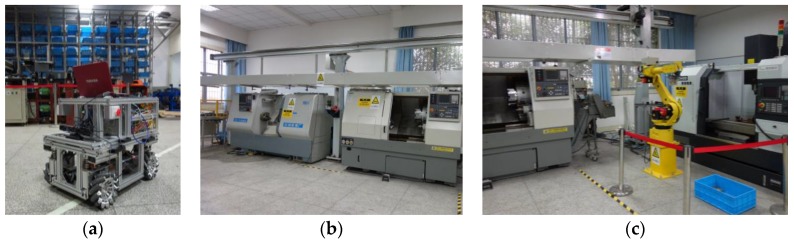
The main equipment of the intelligent manufacturing system. (**a**) Stero warehouse; (**b**) CNC lathes and gantry manipulator; (**c**) industrial robot and milling machine.

**Figure 9:**
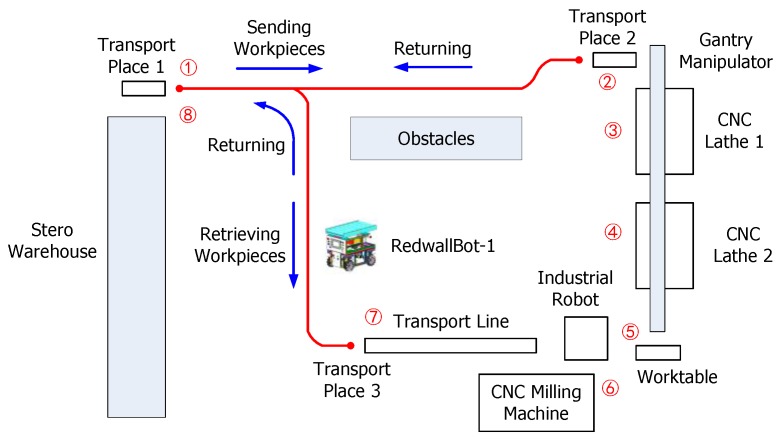
The sequential movement process of the workpieces in the system.

The mobile robot RedwallBot-1 is applied to an intelligent manufacturing system for demonstration, which has several automatic machines including a stero warehouse, a gantry manipulator, two CNC (Computer Numeric Control) lathes, an industrial robot, a CNC milling machine, etc. Figure 8 shows the main equipment and Figure 9 plots the sequential movement process of the workpieces in the system. Rough parts are stored in the stero warehouse initially, and they are carried by the mobile robot to the lathe machining area. A gantry manipulator moves them from machining places to the worktable orderly. After that, a FANUC industrial robot transports them to the CNC milling machine. When the machining operation is terminated, the mobile robot carries the finished products to the stero warehouse again.

## 4. Mobile Robot Localization

It is assumed that the working area of the mobile robot is the ideal horizontal two-dimensional environment. The robot pose at the *k*-th sampling instance is denoted as the status vector **x**(*k*) = (*x_k_*, *y_k_*, *ψ_k_*)^T^. Here, the first two variables determine the central position and *ψ_k_* is the heading angle of the robot in the global coordinate frame *xOy*. Therefore, **x**(*k*) determines the pose transformation of the robot coordinates *x_R_O_R_y_R_* (see Figure 7) relative to *xOy*.

To estimate the robot pose incrementally, an extend Kalman filter (EKF) algorithm [33,34] is used recursively by integrating the data from wheel encoders and the Kinect sensor. In each recursion, both the status prediction and the following measurement update form the frame of estimation. The frequency of the pose estimation is 30 Hz, which is decided by the Kinect sensor.

### 4.1. Status Prediction

Once the mobile robot’s motion function **f**(·) is given accurately, the new pose at the time instance *k* + 1 can be represented as

**x**(*k* + 1) = **f**(**x**(*k*), **u**(*k* + 1), **w**(*k* + 1)) (3)

where **u**(*k* + 1) and **w**(*k* + 1) are the control input and the process noise, respectively. **u**(*k* + 1) is determined by the rotating angles of the wheels. Thus, **u**(*k*) = (*dθ*_1_, *dθ*_2_, *dθ*_3_, *dθ*_4_)^T^. Here, *dθ_i_* means the angular increment of the *i*-th wheel in the sampling interval *dt*, and it is measured by the *i*-th wheel encoder. 

The predicted value of **x**(*k* + 1) is

(4) $${\hat{x}}^{-}(k+1)=\hat{x}(k)+\left[\begin{array}{ccc} \cos{\hat{\psi }}_{k} & -\sin{\hat{\psi }}_{k} & 0\\ \sin{\hat{\psi }}_{k} & \cos{\hat{\psi }}_{k} & 0\\ 0 & 0 & 1 \end{array}\right]\left[\begin{array}{c} v_{x}\\ v_{y}\\ w \end{array}\right]dt,$$

where the hat ˆ above the status vector denotes its estimated value and the additive superscript ^−^ denotes the predicted value.

According to Equation (1), if the coefficient matrix on the right side of the forward kinematics formula is replaced by a coefficient matrix J, then

[vxvyw]=J·[θ˙1θ˙2θ˙3θ˙4], J=πr720[11111−11−1−1d1d−1d1d].

Equation (4) can be represented as

(5) $${\hat{x}}^{-}(k+1)=\hat{x}(k)+\left[\begin{array}{ccc} \cos{\hat{\psi }}_{k} & -\sin{\hat{\psi }}_{k} & 0\\ \sin{\hat{\psi }}_{k} & \cos{\hat{\psi }}_{k} & 0\\ 0 & 0 & 1 \end{array}\right]\cdot J\cdot u(k).$$

The prior covariance matrix P^−^(*k* + 1) of the predicted value of **x**(*k* + 1) is

(6) $$P^{-}(k+1)=\nabla f_{x}P(k)\nabla f_{x}^{T}+\nabla f_{w}Q(k+1)\nabla f_{w}^{T},$$

where P(*k*) and Q(*k* + 1) are the covariance matrices of the pose **x**(*k*) and the process noise **w**(*k* + 1), respectively. The initial values of P(0) and the value Q(*k*) are commonly designated as the diagonal matrices. The superscript T means the matrix transposition. The Jacobian matrices ∇**f**_x_ and ∇**f**_w_ are

$$\nabla f_{x}=\left[\begin{array}{ccc} 1 & 0 & M_{1}\\ 0 & 1 & M_{2}\\ 0 & 0 & 1+M_{3} \end{array}\right],\;\nabla f_{w}=\left[\begin{array}{ccc} \cos{\hat{\psi }}_{k} & -\sin{\hat{\psi }}_{k} & 0\\ \sin{\hat{\psi }}_{k} & \cos{\hat{\psi }}_{k} & 0\\ 0 & 0 & 1 \end{array}\right]\cdot J\cdot I_{4},$$

where I_4_ is a four order identity matrix. The intermediate elements M*_i_* (*i* = 1, 2, 3) in the matrix ∇**f**_x_ are calculated as

$$\left[\begin{array}{c} M_{1}\\ M_{2}\\ M_{3} \end{array}\right]=\left[\begin{array}{ccc} -\sin{\hat{\psi }}_{k} & -\cos{\hat{\psi }}_{k} & 0\\ \cos{\hat{\psi }}_{k} & -\sin{\hat{\psi }}_{k} & 0\\ 0 & 0 & 1 \end{array}\right]\cdot J\cdot u(k).$$

### 4.2. Measurement Update

In this stage, the on-board exteroceptive sensors are commonly used to perceive the environment and then regulate the robot pose according to the residual of measurement. The relationship between the new measurement vector **z**(*k* + 1) and the measurement function **h**(·) is represented as

**z**(*k* + 1) = **h**(**x**(*k* + 1), **v**(*k* + 1)) (7)

where **v**(*k* + 1) is the measurement noise. Equation (7) establishes the connections between **x**(*k* + 1) and **z**(*k* + 1).

A Kinect sensor is used to provide color image and depth image. Both of the two images at the same time instance constitute an RGB-D image. By using the API functions supplied by OpenCV, visual feature extraction and matching are easy to be carried out. First, each RGB-D image is processed to acquire the feature points according to SURF (Speeded Up Robust Feature) algorithm [35] first. Then, the visual odometry method is used to realize relative pose estimation between adjacent image frames. This method includes three steps, i.e., feature matching by using FLANN (Fast Library for Approximate Nearest Neighbors) [36], relative pose determination by combining P*n*P (Perspective-*n*-Point) [37] with ICP (Iterative Closest Point) algorithm [38], and pose optimization by using g^2^o (General Graph Optimization) [39]. Finally, the three-dimensional position increment vector and the three-dimensional posture increment vector could be calculated. Since the work site is horizontal, only the increments along the *x*, *y* directions and the rotating angle around the *z*-axis are needed. Owing to the difference between the installing position of the Kinect sensor and the geometric center of the mobile robot, the increments should be changed into the values corresponding to the robot coordinates. Now, the measurement vector can be described as **z**(*k* + 1) = (*dx_R_*, *dy_R_*, *dψ_R_*)^T^, which is made up of the changes of the position and the heading angle of the mobile robot measured by the Kinect sensor.

According to the EKF algorithm, the predicted measurement vector is

(8) $$\hat{z}(k+1)=\left[\begin{array}{ccc} \cos{\hat{\psi }}_{k} & \sin{\hat{\psi }}_{k} & 0\\ -\sin{\hat{\psi }}_{k} & \cos{\hat{\psi }}_{k} & 0\\ 0 & 0 & 1 \end{array}\right][{\hat{x}}^{-}(k+1)-\hat{x}(k)].$$

Referring to Equation (4), the result is

$$\hat{z}(k+1)=\left[\begin{array}{c} v_{x}\\ v_{y}\\ w \end{array}\right]dt.$$

The residual of measurement, i.e., ***ν***(*k* + 1), is the difference between the measurement vector and the predicted measurement value. It should be noted that the right side of Equation (8) involves the posterior estimation of **x**(*k*). In fact, it is not an exact value and has a probabilistic distribution. An approximation is used to simplify the calculation. The residual of measurement, i.e., ***ν***(*k* + 1), and its approximate covariance matrix is expressed as follows:

(9) $$\nu (k+1)=z(k+1)-\hat{z}(k+1).$$

(10) $$S(k+1)\approx \nabla h_{x}P^{-}(k+1)\nabla h_{x}^{T}+R(k+1).$$

Here, the Jacobian matrix ∇**h**_x_ is

$$\nabla h_{x}=\left[\begin{array}{ccc} \cos{\hat{\psi }}_{k} & \sin{\hat{\psi }}_{k} & 0\\ -\sin{\hat{\psi }}_{k} & \cos{\hat{\psi }}_{k} & 0\\ 0 & 0 & 1 \end{array}\right].$$

The covariance matrix R(*k* + 1) corresponds to the measurement noise **v**(*k* + 1), which relates to the error of the ICP algorithm and the performance of the Kinect sensor. R(*k* + 1) is also designated as a diagonal matrix.

The Kalman gain matrix is calculated as

(11) $$K(k+1)=P^{-}(k+1)\nabla h_{x}^{T}S{\left(k+1\right)}^{-1},$$

where the superscript −1 represents the matrix inverse operation.

Then, the posterior estimation of the robot pose after correction and its covariance matrix are

(12) $$\hat{x}(k+1)={\hat{x}}^{-}(k+1)+K(k+1)\nu (k+1),$$

(13) $$P(k+1)=(I_{3}-K(k+1)\nabla h_{x})P^{-}(k+1)$$

where I_3_ is a three-order identity matrix.

**Figure 10:**
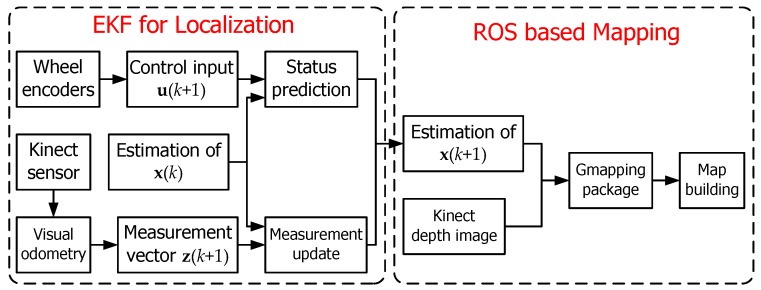
Flow chart of the mobile robot localization and mapping.

According to the EKF algorithm described above and the package of GMapping supplied by ROS, the incremental map of the environment that the mobile robot has explored could be built with the representation of a grid map. Figure 10 shows the flow chart of the mobile robot localization and mapping.

## 5. Experiments and Discussion

### 5.1. Mobility and Stability Tests

The mobile robot RedwallBot-1 was tested on a smooth marble floor. The linear movements were tested first. Once the robot moves an additional 1 m, the error of the linear moving distance was manually measured. Several test results along different directions show that the means and standard deviations of the errors are between 1 and 4 mm, respectively. Therefore, the robot can run in a straight line. The rotational movements were also tested to detect the slippage. The robot rotated at a longitudinal linear velocity and an angular velocity of 0.25 m/s and 0.25 rad/s simultaneously. After the robot rotated once and returned to the starting point, the robot slipped 70~80 mm towards the outside of the circle. This occurred because the wheels on the outside of the mobile robot rotated faster than the wheels on the other side and because the surface of the marble floor was too slick.

**Figure 11:**
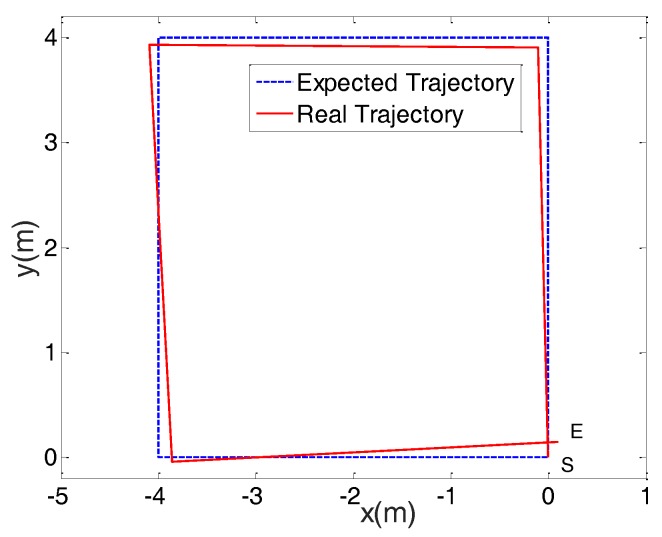
Motion trajectory error when the robot moves along a square.

The serial movements with linear motion and rotation on the spot were implemented to trace a square with a side length of 4 m. The linear velocity and angular velocity were 0.3 m/s and 0.6 rad/s, respectively. Results are shown in Figure 11. When the robot moved from Point S to Point E, the positional errors were 100 mm and 150 mm at two different directions. The errors were mainly caused by slippages when rotating at the corners.

**Figure 12:**
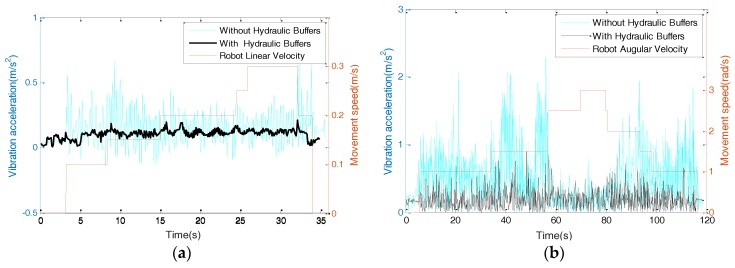
Vibration curves under different moving modes. (**a**) Longitudinal movement; (**b**) rotational movement.

In order to test the vibration and stability of the mobile chassis, a 2-axial accelerometer (ADI ADXL203) was installed on the mobile robot to detect the vertical vibration. The test site was an indoor environment with a smooth marble floor. Figure 12 shows the vibration acceleration curves when the robot moved in different directions with designated velocities. According to comparisons, the vibrations after installing hydraulic buffers were explicitly restrained when the robot moved linearly or turned on the spot. The corresponding vertical slippage distance of each wheel along the cylinder slides was within 10 mm. There is still a high-frequency vibration, which can be weakened by using passively compliant connections between the chassis and the control layers in the next step.

### 5.2. Localization and Mapping Experiments

**Figure 13:**
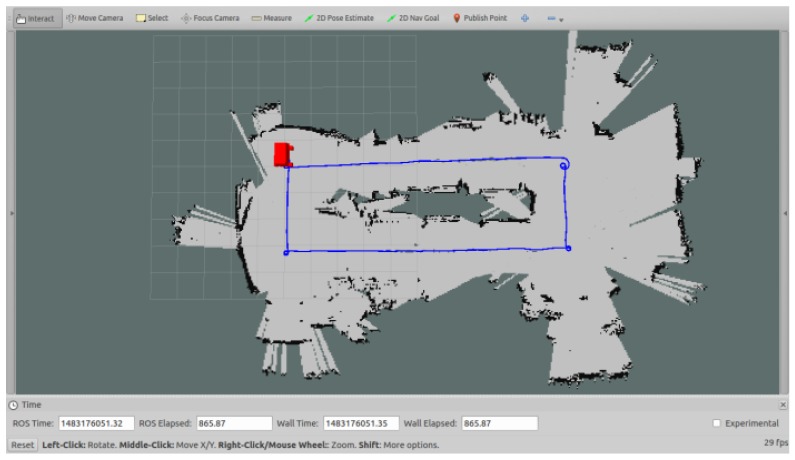
Localization and mapping in the production line.

**Figure 14:**
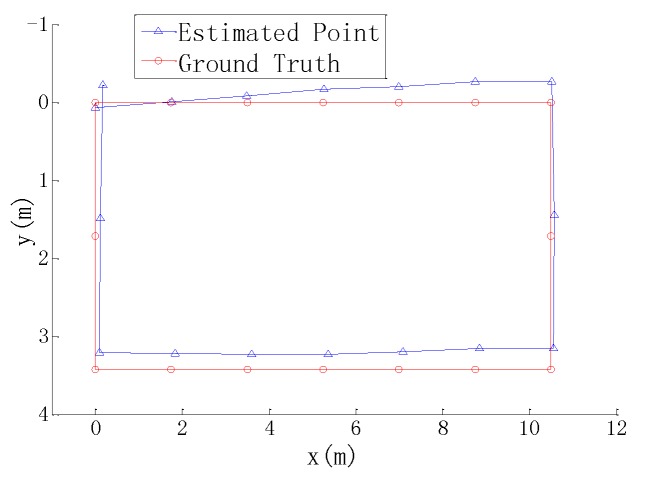
The comparison between estimated and real positions.

To test the performance of localization and mapping, the mobile robot was placed in the intelligent manufacturing production line (see Figure 9). This area was full of equipment and parts. The length and width of the area were about 30 m and 15 m, respectively. The ground was covered by cement with white pebbles. According to the positioning method mentioned in Section 4, the mobile robot used the data of a Kinect sensor and four wheel encoders for localization and mapping. The estimated trace of the mobile robot and the incrementally generated grid map are shown in Figure 13. The mobile robot started from the zero point and moved clockwise along an approximate rectangular trace. Finally, it returned to the start point. Several estimated points and their corresponding practical positions were compared with each other, as shown in Figure 14. The average deviation of the distances between each estimated and real point is 207 mm. Since the Kinect sensor has a limited distance of 4 m and a small field of view, it cannot supply efficient measurements when the environment is open and empty, especially when the robot turns around. In the future research, a laser range finder, an inertial measurement sensor, and a magnetic compass will be integrated to provide more measurement data.

## 6. Conclusions

An omni-directional mobile robot RedwallBot-1 with four Mecanum wheels was developed and tested in this study. The three-layer mechanical design and two-level control system ensure flexible applications and further reorganization. Data from wheel encoders and a Kinect sensor were fused using a simplified extender Kalman filter to localize the mobile robot because only the relative measurements could be obtained. RedwallBot-1 was integrated with an intelligent manufacturing system to convey materials. The preliminary experimental results show that the autonomous localization and mapping method is feasible in industrial fields, with small estimation errors. Future work will focus on improvements in accuracy and reliability of navigation using more on-board sensors. This mobile robot can be used as autonomous conveying equipment as well as a mobile platform for research.

## Figures and Tables

**Table 1:** General specifications of RedwallBot-1.

Description	Quantity
Mobile Body Length	760 mm
Mobile Body Width	500 mm
Mobile Body Height	600 mm
Wheel Diameter	200 mm
Max. Velocity of the Body	1.4 m/s
Max. Rotational Velocity of the Body	3.0 rad/s
Mass of the Mobile Body	80 kg
